# Effects of the COVID-19 pandemic on sexual functioning and activity: a systematic review and meta-analysis

**DOI:** 10.1186/s12889-021-12390-4

**Published:** 2022-01-28

**Authors:** Mojgan Masoudi, Raziyeh Maasoumi, Nicola Luigi Bragazzi

**Affiliations:** 1 Department of Midwifery and Reproductive Health, School of Nursing and Midwifery, Tehran University of Medical Sciences, Tehran, Iran; 2 Nursing and Midwifery Care Research Center, School of Nursing and Midwifery, Tehran University of Medical Sciences, Tehran, Iran; 3 Laboratory for Industrial and Applied Mathematics (LIAM), Department of Mathematics and Statistics, York University, Toronto, ON Canada; 4 Department of Health Sciences (DISSAL), Postgraduate School of Public Health, University of Genoa, Genoa, Italy

**Keywords:** COVID-19 pandemic, Viral outbreak, Sexual functioning and activity, Systematic review and meta-analysis

## Abstract

**Background:**

Since December 2019, when it was first reported in Wuhan, province of Hubei, China, the new virus SARS-CoV-2 has spread rapidly around the world and has become a global pandemic. During the COVID-19 pandemic, due to the public health measures implemented, people’s sexual activity has also been affected. Restrictions on people’s activities, reduced sports activities, economic issues, increased psychological stress, and reduced entertainment have, indeed, dramatically impacted sexual activity and functioning. The purpose of this study was tosystematically identify, collect and summarize the existing body of evidence from published studies on the effects of COVID-19 pandemic on sexual activity and functioning.

**Methods:**

Several scholarly databases, namely MEDLINE (via PubMed interface), Web of Science (WOS), Embase, CINAHL, the Cochrane Library, Scopus, and PsycINFO databases, were mined from December 2019 to the end of January 2021. We utilized a random-effect meta-analytical model to analyze all the data. More in detail, the Standardized Mean Difference (SMD) was used in order to estimate and evaluate the effects of the COVID-19 pandemic on sexual activity and functioning.

**Results:**

Twenty-one studies were included in the present study. In total, 2454 women and 3765 men were evaluated. In the present meta-analysis, sexual functioning and activity were assessed by means of two standardized and reliable tools, namely the “Female Sexual Function Index” (FSFI) and the “International Index of Erectile Function-5 items” (IIEF-5). A total of 5 studies reported the FSFI score before and after the COVID-19 pandemic in female participants. Based on the random-effect model, the SMD was computed to be − 4.26 [95% confidence interval or CI: − 7.26, − 1.25], being statistically significant. A total of 3 studies reported the IIEF-5 score before and after the COVID-19 pandemic in male participants. Based on the random-effect model, the SMD was computed to be − 0.66 [CI 95%: − 0.99, − 0.33], being statistically significant. In the majority of these studies, participants reported a reduction in the number of sexual relations and an increase in the frequency of solo sex activity, especially masturbation, compared to the time prior to the COVID-19 pandemic**.**

**Conclusion:**

The results of the present study showed that COVID-19 related restrictions were correlated with higher rates of sexual dysfunction and reduced sexual activity; however, results of the current meta-analytical study show that this change in sexual functioning was greater in women compared to men. Sex is one of the dimensions of every person’s life; therefore, researchers should identify the factors that lead to sexual dysfunction due to COVID-19 pandemic in their community. In this regard, sexologists should design and implement effective programs to reduce the heterogeneous causes affecting sexual functioning, given the psychological strain that the COVID-19 pandemic puts on individuals.

**Supplementary Information:**

The online version contains supplementary material available at 10.1186/s12889-021-12390-4.

## Background

In late December 2019, the new virus “SevereAcute Respiratory Syndrome-related Coronavirus type 2” (SARS-CoV-2) was first reported in Wuhan, province of Hubei, China. Since then, it has spread rapidly around the world and has become a global pandemic [1, 2], representing a serious public health challenge [3]. SARS-CoV-2 is the infectious agent responsible for the “Coronavirus Disease 2019” (COVID-19) pandemic, which is imposing a dramatically relevant toll of infections and deaths, with more than five million people who have lost their lives and more than 250 million infected cases in the world as of November 22, 2021 [4]. Due to the lack of immediately available specific and effective treatment methods and vaccines, which have been introduced later [5, 6], policy- and decision-makers have been working to prevent the spread of the disease by implementing non-pharmaceutical interventions (NPIs), such as enhanced public health and hygiene practices and increasing diagnostic testing capacity [7, 8].

The major goal of these programs was to minimize the conditions that would have facilitated disease transmission [9]. Social distancing and self-isolation have been implemented in many countries to prevent or, at least, mitigate against the spread of COVID-19 [10, 11]. If, on the one hand, these interventions have contributed to saving lives, on the other hand, these policies have profoundly affected the societal and psychological aspects of people’s daily routine and have changed many of their habits [12, 13]. Governments have been closely monitoring the implementation of these measures, and, in the meantime, have been trying to mitigate against the societal burden generated by the COVID-19 related public health interventions [14].

During the COVID-19 pandemic, many people have been, indeed, experiencing anxiety, depression, panic, as well as economic-financial problems, such as unemployment, increased poverty, and declining incomes and earnings [14–16], that have affected people’s sexual activity [17]. Restrictions on people’s activities, reduced sports activities, economic issues, increased psychological stress, and reduced entertainment have dramatically impacted sexual activity and functioning [18]. In this regard, it can be claimed that, under unusual circumstances, people’s sexual behaviors tend to change dramatically [19]. Because people are forced to be in close proximity or away from their sexual partners, this may emotionally influence their relationship, which will consequently affect their sexual behavior [20].

Sexual dysfunctions are a heterogeneous group of disorders that are typically characterized by a clinically significant disturbance/impairment in a person’s ability to respond sexually or to experience sexual pleasure [20]. Specific sexual dysfunctions include hypoactive sexual desire disorder, erectile dysfunction (ED), orgasmic and ejaculatory disorder, and genito-pelvic penetration pain disorder [20, 21]. Sexual dysfunctions are highly prevalent, affecting about 43% of women and 31% of men [22]. Hypoactive sexual desire disorder has been reported in approximately 30% of women and 15% of men in population-based studies, and is associated with a wide variety of medical and psychologic causes [22]. Also, concerns around low sexual desire are one of the most widespread sexual problems adults can face. ED is another most common male sexual health concern, affecting 13–28% of men, aged 40–80 years. While no data has been reported regarding the relationship between COVID-19 and the additional risk of developing ED, men are thought to be at greater risk of having serious complications due to the COVID-19 pandemic. Moreover, those who are traditionally at risk for ED would be at greater risk during the pandemic [23, 24]. Home confinement during the COVID-19 outbreak, combined with the psychological stress of living in a pandemic may amplify the already existing sexual dysfunction, as well as sexual desire discrepancy [21]. To the best of our knowledge, there have been no systematic reviews with meta-analyses so far on how many people have actually experienced changes in their sexual behavior since the start of the COVID-19 pandemic [25]. Based on the available literature on the three main areas of research (sexual desire, arousal, and orgasm), findings are rather contrasting. Most participants reported that sexual desire varied - increased or decreased - during the lockdown, compared to a pre-lockdown baseline. For instance, results of Ballester-Arnal et al.’s study (2020) in Spain show that 35.9% of participants stated that they had greater sexual desire during confinement; on the other hand, 34.9% reported a downward sexual desire [26]. Approximately, 18.2% of men and 26.4% of women reported a decrease in sexual desire in Panzeri et al.’s study in Italy (2020) [27]. On the contrary, results of Li et al.’s study (2020) in China show that 61% of the sample reported that their sexual desire have not varied. In another study, 25% of participants reported less sexual desire and only 14% (18% of men and 8% of women) experienced an increase in their sexual desire [28]. Also, in most studies, the effects of the COVID-19 pandemic on sexual functioning of men and women were found to be different; for example, results of Wignall et al.’s study (2021) in UK show that men reported higher sexual desire levels compared to that of women both before and during the lockdown. Results also show that women reported a significant reduction in the levels of sexual desire during the lockdown [29].

Sexual activity is an umbrella term that can refer to various forms of sexual behaviors and expressions [30]. While ‘sexual activity’ usually incorporates sexual intercourse, it is clear that it can also encompass emotional intimacy, close companionship, flirting, affection, petting, hugging, kissing, desire, and masturbation [31–33]. Overall, the majority of research in this area has focused on partnered sexual activity [34].

Critical situations such as the COVID-19 pandemic can affect the frequency and duration of sexual intercourse, as well as quality of sexual activity [21, 22]; on the other hand, sexual intercourse could be a major risk of contagion [35] because sexual intercourse requires close physical contact, and SARS-CoV-2 is very easily transmitted with this level of closeness [36]. Conversely, healthy, safe, and frequent sexual activity might attenuate the negative psychological effects associated with the infection [35]. Results of various studies on the impact of the COVID-19 pandemic on sexual activity are very different [36–38]. Some of these results show that there may be gender-specific differences in the way the lockdown influenced the frequency of sexual intercourse [38].

Since the beginning of the COVID-19 pandemic, researchers have been studying the impact of this disease and the policies implemented to contain the outbreak on various societal phenomena, including sexual activity and functioning. Awareness of the findings of these studies can help public health policy- and decision-makers identify effective causes of decreased/impaired quality of sexual activity, and design and provide effective programs to improve it. Therefore, given the contrasting findings of the literature, the purpose of this study was to systematically identify, collect and summarize the existing body of evidence from published studies on the effect of the COVID-19 pandemic on sexual activity and functioning by means of standardized and reliable tools.

## Methods

We reported the findings of this review in accordance with the “Preferred Reporting Items for Systematic Reviews and Meta-Analyses” (PRISMA) guidelines [39] (Supplementary 1).

### Search strategy

From December 2019 to the end of January 2021, we searched MEDLINE (via its PubMed interface), Web of Science (WOS), Embase, CINAHL, the Cochrane Library, Scopus, and PsycINFO databases. The search was conducted by two authors independently. The authors first found the relevant keywords based on the purpose of the study and, then, used the following search strategy utilizing Boolean connectors:

(“sexual health” OR “sexual function” OR “sexuality” OR “sexual development” OR “sexual disorders” OR “sexual dysfunction” OR “sexual and gender disorders” OR “sexual behavior” OR “sexual activity” OR “sexual activities” OR “sexual quality of life” OR “desire” OR “anorgasmia” OR “erectile dysfunction” OR “libido” OR “ejaculation” OR “impotence” OR “hypersexuality” OR “sexual impulse control” OR “arousal” OR “vaginismus” OR “orgasm” OR “paraphilia” OR “hyposexuality” OR “priapism” OR “sexual misconduct” OR “sexual deviation” OR “sexual motivation” OR “sexual dissatisfaction” OR “sexual intention”) AND (“2019-nCoV” OR “covid-19” OR “SARS-CoV-2” OR “severe acute respiratory syndrome coronavirus 2” OR “severe acute respiratory syndrome coronavirus” OR “corona-virus” OR “COVID” OR “coronavirus” OR” SARS-CoV” OR “MERS-CoV” OR “SARS-CoV-2″ OR “COVID-2019″ OR” 2019 Novel Coronavirus”).

Google Scholar was also mined to increase the chance of finding potentially relevant studies. The reference list of these potentially eligible investigations was also searched. Records identified from database searches were exported to EndNote Version 9 (Clarivate Analytics). The disagreement in study retrieval between the two authors was resolved involving a third author, if necessary, or through discussion until the agreement was achieved.

### Study selection criteria

#### Inclusion criteria for selecting relevant studies

Studies published in English, studies whose findings were transparent, studies published in peer-reviewed journals, studies whose full text was available, studies in which participants were healthy (without mental illnesses or other disorders), and studies in which participants were clearly stratified according to their gender (male or female) were considered. Moreover, in this systematic review and meta-analysis only heterosexual participants were included.

#### Criteria for excluding not relevant studies

Studies published in non-English language journals, studies whose full text was not available, studies that lacked clear and interpretable findings, studies in which participants had a specific illness, and studies on LGBT+ people, including lesbian, gay, bisexual, transgender, and other gender diverse individuals, were excluded. If in one study there were LGBT+ people, we assessed data only from heterosexual participants/populations.

### Data extraction

Two reviewers independently extracted data from selected studies. The disagreement between the two authors was resolved involving a third author, if necessary, or through discussion until the agreement was achieved.

They collected data and extracted: author’s first name, year of publication, location and setting, number of participants (sample size), gender percentage of participants, design of the study, study collection method, data collection tool, mean age of participants, indicator used and the most important findings of the study.

Sexual activity and functioning were assessed by various scales and questionnaires: among the different tools, the “Female Sexual Function Index and International Index” (FSFI), the “Premature Ejaculation Diagnostic Tool” (PEDT), and the “International Index of Erectile Function-5 items” (IIEF-5) were the most utilized in the included studies.

### Methodological quality assessment

To assess the quality of the methodology of the included studies, a checklist with eight items was used. This checklist was devised and employed in a study by Li et al. in a recently published COVID-19-related meta-analysis study [40]. Scores from 0 to 2 (0 = unreported, 1 = reported but insufficient, and 2 = sufficient) were used to answer to each item.

### Statistical analysis

The Standardized Mean Difference (SMD) (Hedges’ g) [41] was used in order to estimate and evaluate the effects of the COVID-19 pandemic on sexual activity and functioning. All effect size estimates were reported with their computed 95% confidence interval (CI). The I^2^ statistics was used to evaluate the amount of heterogeneity among the studies [42]. In the present meta-analysis, we utilized the random-effect model to analyze all the data. Data were analyzed using the Review Manager (RevMan) Ver.5.3 software. *P*-value < 0.05 was considered significant. Considering that the data were enough only for two indicators (namely, the FSFI and IIEF-5), we thus were able to perform a meta-analysis for the studies which utilized these tools. However, due to the fact that for the two indicators (FSFI and IIEF-5) synthesized in the present meta-analysis, the number of studies was less than ten, it was not possible to assess the publication bias.

## Results

### Study selection

**Fig. 1:**
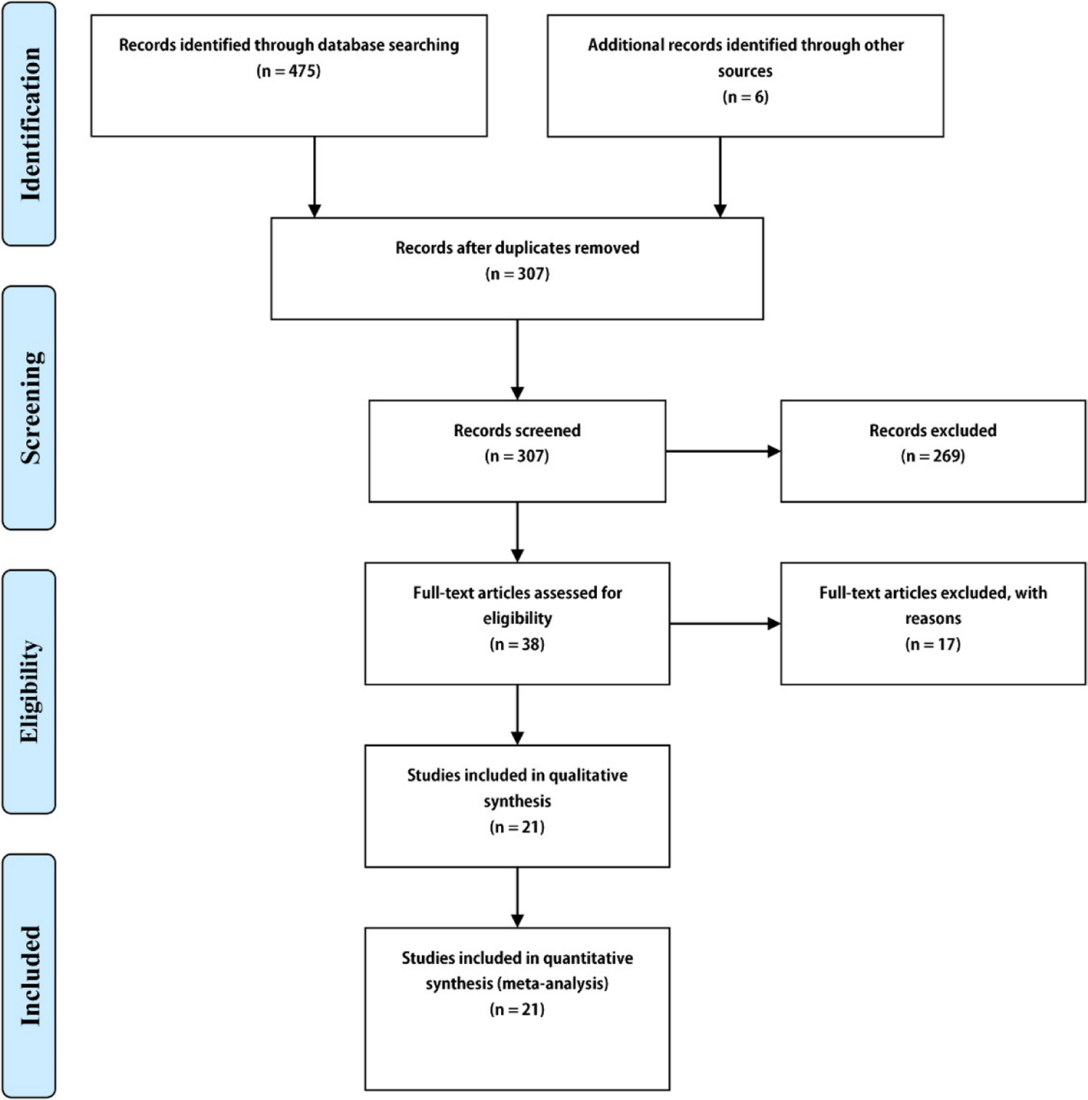
Flow diagram of the study selection process

The initial databases search found 481 studies. After removing duplicate studies, a total of 307 records were retained. By reviewing the title and abstract, 269 studies were excluded. After assessing the full text of 38 articles, 17 articles were deleted for various reasons. Finally, 21 articles were included based on the above-mentioned inclusion criteria [26, 28, 37, 43–60]. All these studies were investigations with convenience samples that reported data at two time points. The study selection process is pictorially shown in Fig. 1.

### Main findings of the included studies

Table 1 shows the most important characteristics and findings of the 21 studies included. All studies used online questionnaires or social media to collect data due to the impossibility of having face-to-face interactions because of the COVID-19 related restrictions. In total, 2454 women and 3765 men were evaluated. FSFI and IEEF-5 were the two measures used in the present meta-analysis to assess sexual activity and functioning.

**Table 1:** Characteristics of included studies

First author	Year	Country	Data collection method	Instrument	Number of participants	Age	Main finding
Li	2020	China	Online survey	Self-management surveys via social media platforms	459 (270 men and 189 women)	15–45 Years	- 44% decrease in the number of sexual partners − 37% of participants reported a decrease in sexual frequency. -Rapid reduction in risky sexual behavior in individuals with risky sexual experiences − 25% reduction in sexual desire - Increased sexual desire in 18% of men and 8% of women - decrease in the frequency of sexual activities in 36% of married men and 28% of married women - reduction in sexual satisfaction in 32% of men and 39% of women
Yuksel	2020	Turkey	Online survey	FSFI	58 Women	27.6 ± 4.4	- Increased average frequency of sexual intercourse (2.4 vs 1.9, P = 0.001). Participants had significantly better FSFI scores before the pandemic compared with scores during the pandemic (20.52 vs 17.56, *P* = 0.001).
Fang	2020	China	Online survey	IIEF-5, PEDT	251 Men	28 (24–35)	−8.4% reported a deteriorated erectile function, 8.5% reported a decrease in ejaculation control - Decreased 31.9% IIEF-5 scores and 17.9% increased PEDT scores - Decreased frequency of sexual life - No changes in their erectile function
Samir Omar	2021	Egypt	Online survey	IIEF-5, FSFI	696 (479 females and 217 males)	25–35	- Higher sexual satisfaction before lockdown −70.5% males and 56.2% females satisfied with their sexual performance −68.2% male no erectile dysfunction −97.3% females scored 26.5 on the FSFI
Jacob	2020	The United Kingdom	Online survey	Self-made questionnaire	868 (men and women)	25–34	−39.9% of the persons reported sexual activity at least once per week. - prevalence of sexual activity was lower than 40%. - sexual activities higher in men than in women
Mahanty	2020	India	Online survey	Self-made questionnaire	262(men and women)	NA	-Frequency of sexual activities higher in women than man. - In women lower frequency of watching porn
Ballester-Arnal	2020	Spain	Online survey	Self-made questionnaire	1448 (471 men and 977 women)	18–60	−37.9% reported worsening of their sexual life − 35.9% stated that they had a higher sexual desire during confinement, 34.9% had a lower desire and 29.1% unchanged. - Men report higher traditional masturbation and online sexual activity than women
Cem Bulut	2020	Turkey	Online survey	IES-R, IIEF-5	359 men	NA	-Decreased sexual function in healthcare professionals.
Baran	2020	Turkey	Online survey	IIEF-5	536 men	38.6 ± 10.3	- Decrease in sexual desire in 25% of the participants
Kaya	2020	Turkey	Telephone interview	FSFI	15 women	33.3 ± 5.6	- Decreased frequency of sexual intercourse and sexual satisfaction
Fuchs	2020	Poland	Online survey	FSFI	764 women	25.1 ± 4.3	- Decreased frequency of sexual intercourse −3.2% women feared that COVID-19 could be transmitted during sexual activity
Yasir Arafat	2020	Bangladesh, India & Nepal)	Online survey	Self-made questionnaire	120 (93 men, 26 women and 1 person, not to say)	35.42 ± 5.73	−76.7% of respondents had sexual intercourse with their spouse 1 to 5 times a week before COVID-19 −45% of the respondents said that the COVID-19 had impact on their sexual activity. − 72.5% of respondents reported after COVID-19, to have sexual intercourse 1 to 5 times a week.
Schiavi	2020	Italy	E-mail	FSFI, FSDS	89 women	39 (28–50)	-Sexual intercourses decreased significantly from 6.3 to 2.3
Li	2020	China	Social media	WeChat or Weibo	967 (541 men and 426 women)	26.6 ± 4.86	−22% reported a decrease in sexual desire - 41% experienced a decrease in the sexual intercourse frequency - 30% reported an increase in the frequency of masturbation −10% reported a decrease in risky sexual behavior
Feng	2020	China	Online survey	Self-made questionnaire	284 (134 men and 150 women)	18–44	−17.6% felt the number of sexual partners has reduced. −43.3% reported decreased sexual frequency and 44 reported an increase with those before the COVID-19. - 25.0% of the participants experienced reduction in sexual desire, and 19.0% experienced an increase. −21.5% suggested that sexual satisfaction has reduced. − 43.3% reported a decrease in sexual frequency.
Luetke	2020	USA	Online survey	Self-made questionnaire	2261 (1119 men, 1142 women)	NA	- Decreased frequency of sexual intercourse -Decreased solo masturbation -Decreased partnered masturbation
Karagöz	2020	Turkey	Online survey	IIEF-5, FSFI	245 volunteers (148 men and 97 women)	35.9 ± 6.9	-Increase in solitary sexual activity (masturbation or watching sexual content videos - Decreased frequency of sexual activities -Decreased sexual satisfaction levels
De Rose	2021	Italy	Online survey	IIEF-5, FSFI	544 (260 men, 284 women)	31	−65.3% reported a decreased sexual desire, in health care workers it waas lower than in non- Health care Workers - 91.2% reported a decrease in Satisfaction sexual, in health care workers this was lower than in non- Health care Workers
Coombe	2020	Australia	Online survey	Online survey	920 (247 men, 673 women) We excluded men who have sex with men and women who have sex with women (40 participants)	NA	- Sex activities in 2019 were higher than during lockdown -Increase in using sex toys
Cocci	2020	Italy	Online survey	Self-made questionnaire	1515 men and women	21	- 15.91% no sexual desire and 4.22% less sexual desire than before COVID-19 - 60.79% see porn movies during the COVID-19 - 53.53% no sexual satisfaction
Karsiyakali	2020	Turkey	Online survey	Self-made questionnaire, FSFI, IIEF-5	1356 (671 men and 685 women)	33.16 ± 8.31	-Decrease in the mean number of sexual activity from 1.86 ± 1.67 to 1.35 ± 2.04 after COVID- 19 -Increase in masturbation frequency from 1.25 ± 1.63 per week to 1.57 ± 2.11 −31.5% participants reported reduction in the number of weekly sexual Intercourses

FSFI: Female Sexual Function Index, IIEF-5: International Index of Erectile Function-5 items scores, PEDT: Premature ejaculation diagnostic tool scores, IES-R: Impact of Event Scale-Revised, NA: Not applicable

#### Quality assessment

**Fig. 2:**
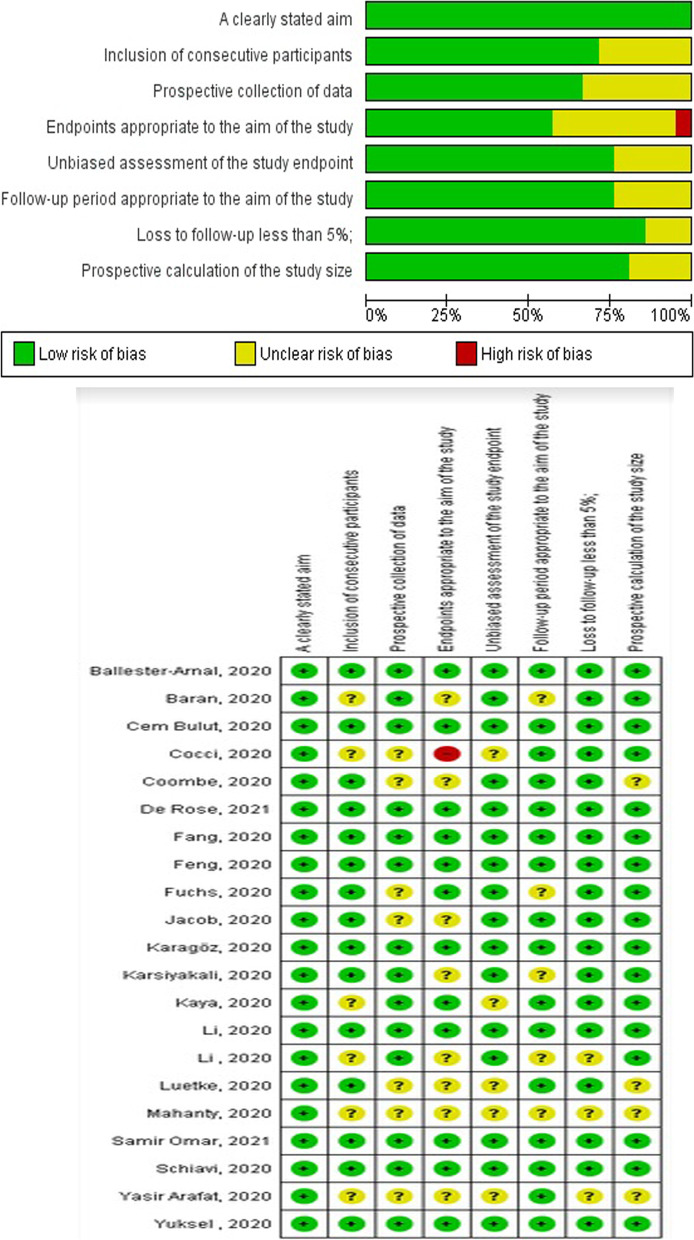
The quality assessment of the studies included in the present systematic review and meta-analysis

Figure 2 and Table 2 show the quality appraisal of the methodology of the included studies.

**Table 2:** The quality appraisal of included studies

First author	Year	Q1	Q2	Q3	Q4	Q5	Q6	Q7	Q8	Score
Li	2020	2	1	2	1	2	1	1	2	14
Yuksel	2020	2	2	2	2	2	2	2	2	16
Fang	2020	2	2	2	2	2	2	2	2	16
Samir Omar	2021	2	2	2	2	2	2	2	2	16
Jacob	2020	2	2	1	1	2	2	2	2	14
Mahanty	2020	2	1	1	1	1	1	1	1	9
Ballester-Arnal	2020	2	2	2	2	2	2	2	2	16
Cem Bulut	2020	2	2	2	2	2	2	2	2	16
Baran	2020	2	1	2	1	2	1	2	2	13
Kaya	2020	2	1	2	2	1	2	2	2	14
Fuchs	2020	2	2	1	2	2	1	2	2	14
Yasir Arafat	2020	2	1	1	1	1	2	1	1	10
Schiavi	2020	2	2	2	2	2	2	2	2	16
Li	2020	2	2	2	2	2	2	2	2	16
Feng	2020	2	2	2	2	2	2	2	2	16
Luetke	2020	2	2	1	1	1	2	2	1	12
Karagöz	2020	2	2	2	2	2	2	2	2	16
De Rose	2021	2	2	2	2	2	2	2	2	16
Coombe	2020	2	2	1	1	2	2	2	1	13
Cocci	2020	2	1	1	2	0	2	2	2	12
Karsiyakali	2020	2	2	2	1	2	1	2	2	14

#### The effect of the COVID-19 pandemic on the FSFI score

**Fig. 3:**
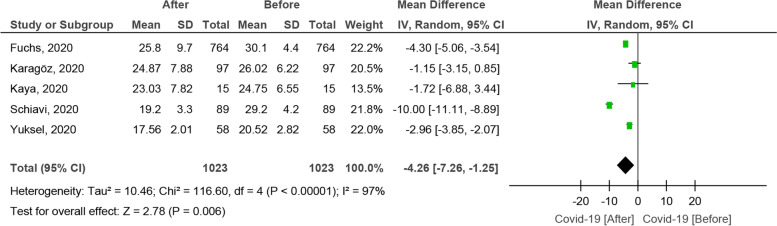
The effect of COVID-19 on the FSFI score

**Fig. 4:**
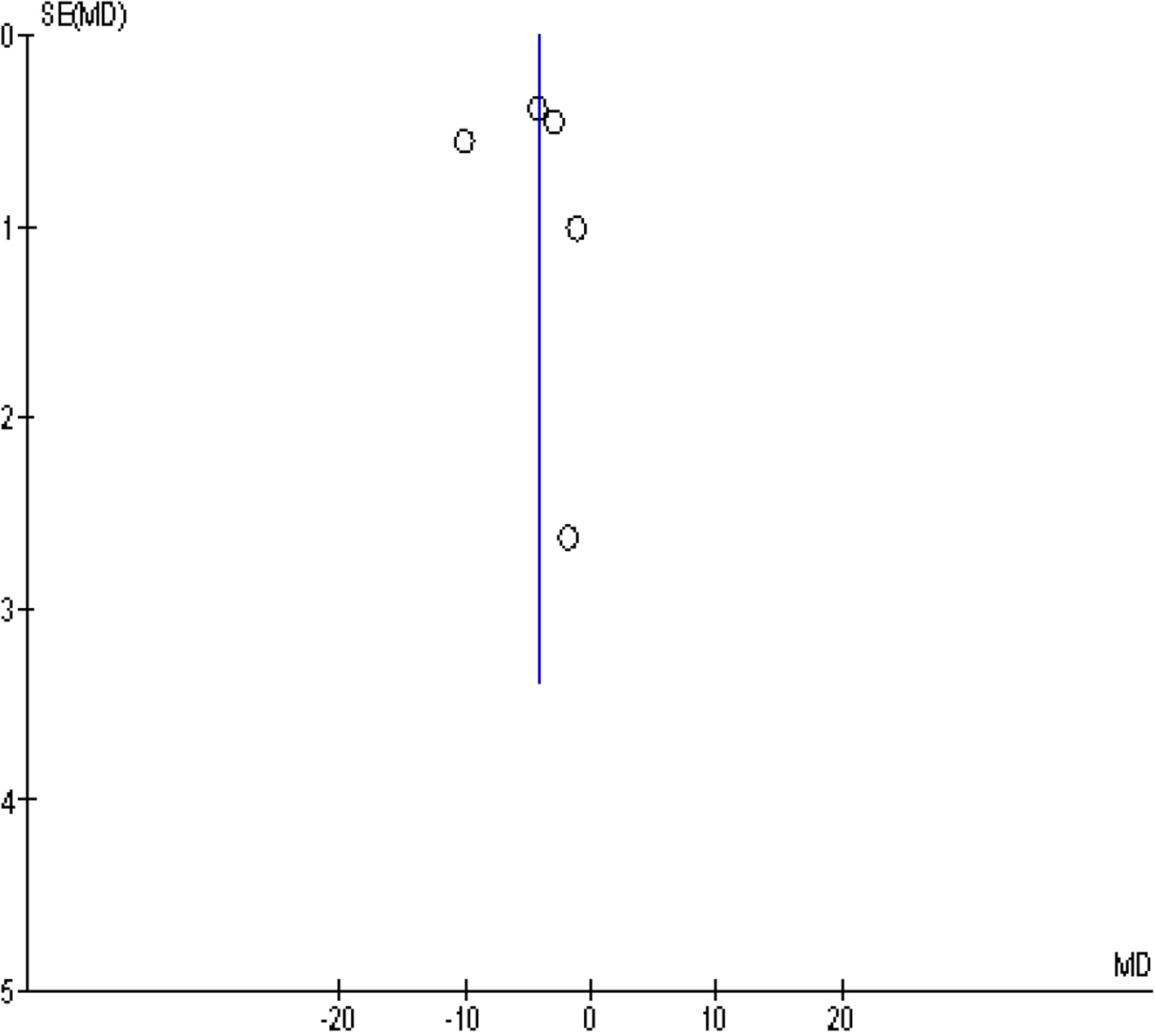
The funnel plot

A total of 5 studies reported the FSFI score before and after the COVID-19 pandemic in female participants [51, 52, 54, 59, 60]. Based on the random-effect model, the SMD was computed to be − 4.26 [CI 95%: − 7.26, − 1.25], being statistically significant (Fig. 3). The funnelplot is shown in Fig. 4.

#### The effect of the COVID-19 pandemic on the IIEF-5 score

**Fig. 5:**
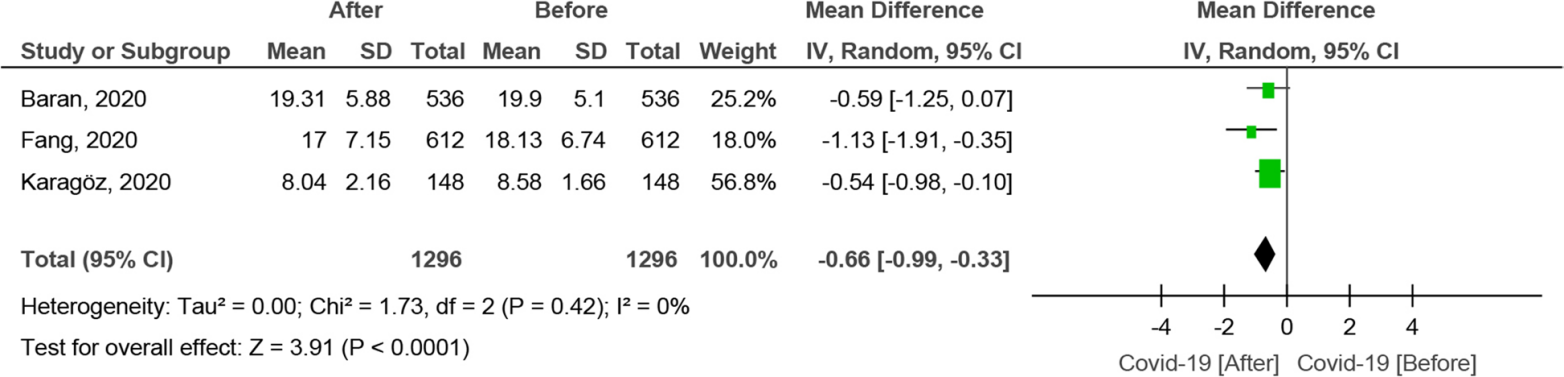
The effect of COVID-19 on the IIEF-5 score

**Fig. 6:**
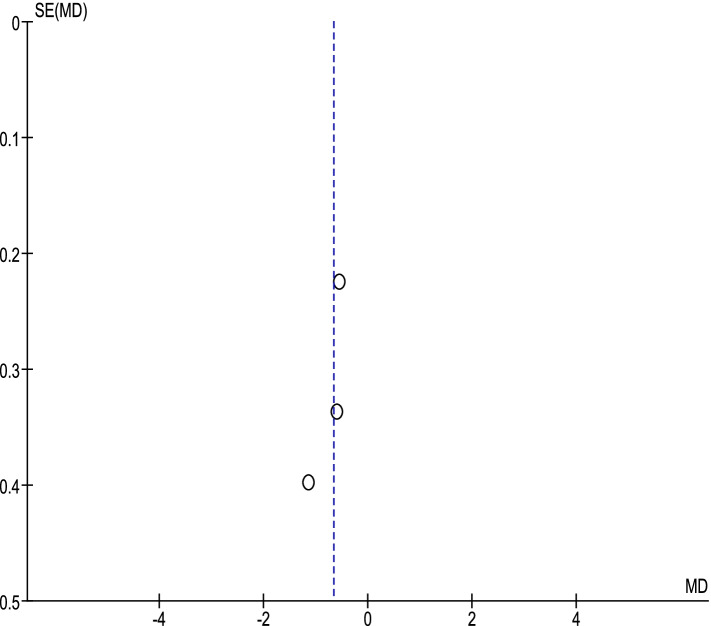
The funnel plot

A total of 3 studies reported the IIEF-5 score before and after the COVID-19 pandemic in male participants [44, 50, 52]. Based on the random-effect model, the SMD was computed to be − 0.66 [CI 95%: − 0.99, − 0.33], being statistically significant (Fig. 5). The funnel plot is shown in Fig. 6.

#### The effect of the COVID-19 pandemic on the PEDT score

Only 1 study reported the PEDT score and, as such, it was not possible to perform any meta-analysis.

#### Factors associated with sexual activity and functioning during the COVID-19 pandemic

**Fig. 7:**
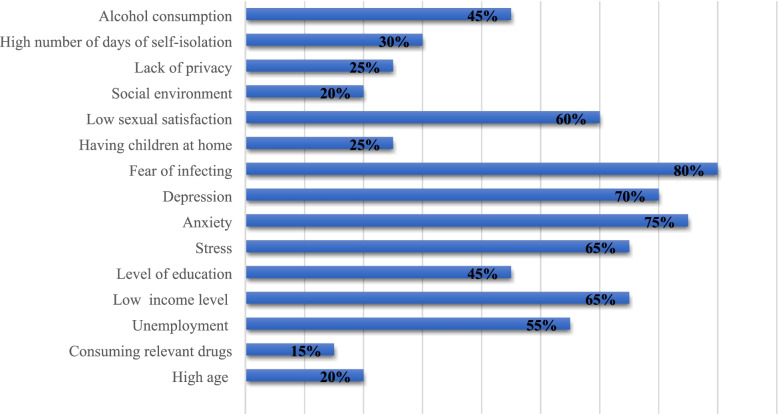
The major factors influencing the participant’s sexual activity and functioning in the included studies

Based on the findings of the included studies, the major factors that influenced participants’ sexual activity and functioning are shown in Fig. 7. According to the data of included studies, various factors have affected the reduction of sexual activity. The factors that have been reported in various studies are expressed in percentages.

#### The effects of the COVID-19 pandemic on sexual activity and functioning

In 17 of the 21 studies included in this review, the effects of the COVID-19 pandemic on sexual activity were investigated. Only in 5 studies [45, 54, 55, 60, 61], the mean number of sexual relationships per week was compared before and during the COVID-19 pandemic. In other studies, prevalence frequency of sexual intercourse (weekly, monthly, and yearly) was compared before and during the pandemic, or the prevalence of changes experienced in frequency of sexual intercourse by participants during the pandemic were reported. Based on the findings of the included studies, in the majority of these studies (15 study) participants reported a reduction in the number of sexual relationships during the pandemic compared to before the COVID-19 pandemic. Also, in most of the included studies (11 studies), the reduction in sexual intercourse was significant. Only in one study, a significant increase in the frequency of sexual intercourse was reported during the COVID-19 pandemic compared to that of the pre-pandemic period [26]. Meanwhile, in two studies, gender-specific differences in the frequency of sexual intercourse during COVID-19 pandemic were significant. In the first study, reported frequency of sexual intercourse in female had been improved as compared to that of male [58]. In the second study, however, the mean number of sexual activities in men was higher than in women [52]. Based on the findings of this systematic review, masturbation and other types of solo sex activity such as online sex and viewing pornography had increased during the COVID-19 pandemic. In most studies, decrease in the frequency of sexual intercourse was associated with a significant increase in the frequency of solo sex activity, especially masturbation. In a number of studies, significant gender-specific differences were found in the frequency and type of solo sex activity. For example, according to the findings of the study of Karagoz et al. (2020), a significant difference (*p* = 0.022) between men and women (12.8%of males versus 4.1% of females) in increase of solitary sexual activity (masturbation or watching sexual content videos, etc.) during the COVID-19 pandemic was reported [52]. Also, the findings of the study of Arnal et al. (2020) showed statistically significant differences according to gender as men reported higher percentages for traditional masturbation without using sex toys (*p* < 0.001) and online sexual activity (p < 0.001), both with a moderate effect size; and women for masturbation using sex toys (p < 0.001) [26]. Finally, in Mahanty. et al’s study (2020), frequency of watching porn in female respondents was significantly higher compared to male respondents [57].

Moreover, participants reported a decrease in the number of sexual partners, sex without marriage (with a girl/boyfriend), and risky sexual behavior. It should be noted that these raw data and estimates were very heterogeneous, and we could not combine it with specific statistical methods; therefore, we were not able to perform a meta-analysis. Accordingly, these findings have been only qualitatively included and assessed in this study, showing the changes in various sexual activity domains before and during the quarantine.

## Discussion

This study investigated the effects of the COVID-19 pandemic on sexual activity and functioning in women and men.

Natural hazards and crises, as well as wars and diseases, can disrupt societal and individual functions [61, 62]. For instance, after earthquakes, the frequency and the quality of sexual behavior have been significantly decreased [63–65]. One of the factors explaining the impaired desire and the reduction in sexual activity is a change in the psychological status of individuals [66]. In the present systematic and meta-analysis, stress, anxiety, and depression were the psychological factors investigated in the included studies that can be considered as possible causes of sexual dysfunction. In this regard, findings of this study showed that the participants’ sexual behavior was decreased among women and men.

After the implementation of COVID-19 related restrictions in many countries, people’s quality of life including sexual behavior with their partners has dramatically changed [67, 68]. Previous studies have shown that natural disasters and wars negatively affect sexual functioning; for example, a study that investigated sexual behavior after a tsunami has reported that 30% of 1093 participants were suffering from sexual dysfunction [33].

In the included studies, the fear of contracting and/or transmitting COVID-19 had the greatest effect on the occurrence of sexual dysfunction. COVID-19 is rarely transmitted through sexual intercourse [69]. Contact with droplets in the mouth, nose and saliva of an infected person can contribute to the transmission of COVID-19 [70]. For this reason, many people cited the fear of transmission of the disease from their sexual partner as the reason for their decreased sexual desire. Asymptomatic infected cases can easily spread the disease to others [71]. Some participants stated that they refused to have sex because they did not know if their sexual partner was ill or not.

Economic activities and various occupations have been affected by the COVID-19 pandemic [7]. Due to social restrictions imposed by governments, many people are unemployed and many people are forced to work fewer hours [72]. Fear of unemployment, inability to pay for living expenses have resulted in higher stress, anxiety and depression levels in people [73]. According to the findings of some studies, low income and socio-economic status (SES) are associated with sexual dysfunction [74, 75]. During the COVID-19 pandemic, men have been forced to spend more time at home due to social constraints and this, together with increased stress levels due to unemployment and sedentary lifestyle, can result in decreased sexual desire or violence against women [57].

In studies in which participants worked in the health sector, they were more likely to suffer from sexual dysfunction than other people working in other occupations [76]. Excessive work-related fatigue and stress, fear of getting sick, and high work-load with increased working hours due to lack of manpower in COVID-19-related service centers caused them to sleep less hours at home [77]. Their quality of life had significantly decreased and they had problems with their partners [78].

Also, the findings of this study showed that higher-educated people experienced a significant reduction in sexual activity. In the included studies, women with higher levels of education were more likely to have less sexual activity than women with lower levels of education. Participants with higher education had more information about COVID-19 and therefore were more afraid of transmitting the disease to their sexual partners than those who were less aware of the disease [79]. According to the findings of some studies, participants with higher levels of education were more likely to experience COVID-19 induced anxiety, stress, and depression [80] and social constraints made them more concerned about losing their job [81]. Furthermore, in the included studies, young health care workers with a higher level of education who were also alone showed no difference in working habits during the COVID-19 induced lockdown and experienced a significantly decrease in their sexual desire compared to other participants [48].

After imposing social restrictions, because of the psychological effects generated by the COVID-19 pandemic, participants’ tendency to consume alcohol increased [82]. Fear and misinformation have led to the belief that alcohol consumption can kill the virus, and some people have turned to alcohol [83]. Alcohol consumption during the COVID-19 outbreak can increase body’s vulnerability, although the WHO has denied this rumor [84]. Alcohol uptake is one of the predisposing factors for developing sexual disorders [85]. ED, decreased libido and sexual dissatisfaction are commonly reported [86]. In addition to decreased sexual desire, there is also an increased prevalence rate of violence and high-risk sexual behaviors [52, 87].

Quarantine and self-isolation during the COVID-19 outbreak have made it difficult for people to travel and to leave their house [46]. With the implementation of these policies, spouses and children are more at home and there is less room for cultivating private and intimate relationships. Worrying about having children in these particularly difficult circumstances can cause stress and anxiety and reduce the frequency of sexual activities [52].

In the studies included in the present systematic review, sexual dysfunction and decreased sexual activity were more common among older participants. Aging in both sexes is naturally associated with physical and physiological changes [88]. Sometimes, these changes affect a person’s ability to enjoy sexual pleasure. Men often experience impotence as they get older, which includes losing the ability to get a proper erection for sexual intercourse [89]. Among older women, fear of not reaching orgasm during sexual intercourse is a commonly reported concern [90]. One of the major concerns of older people is the higher morbidity and mortality of COVID-19 [91]. These people tend to stay at home longer due to fear of infection, and their social activities are much more limited and their mental problems are more likely to worsen, if already present [92].

In this study, we found that the FSFI score in women before and after the COVID-19 pandemic decreased significantly. Studies show that being in a critical situation for women can reduce their social activities and can cause as well sexual dysfunction [52, 60, 64]. Moreover; our results also showed that the IIEF-5 score decreased in men and this decrease was as well statistically significant. The decrease in sexual activity and desire was, however, higher among females than among men, and this could reflect a gender-specific difference, as found by other studies. According to these investigations, this difference can be due to sex- and gender-related variables, in terms of physical and mental characteristics of men and women, and, as such, the response to exposure to the COVID-19 pandemic can be different and differently affect sexual activity [69]. One of the reasons for such differences could be that women are more willing to respond transparently concerning their sexual status during the COVID-19 pandemic than men [66]. In a study conducted to find the causes of this difference, the majority of women declared that the reason might be associated with isolation from their partner (41.5%), 39.3% felt lack of desire caused by stress, and 16% had misunderstandings with their partners [51]. Also, sexual stress was significantly greater in females than males [58]. Furthermore, chronic stress increases cortisol levels especially in women, leading to sexual dysfunction, in particular decreased sexual arousal [64].

Based on the findings of the systematic review and of the studies included in this paper, there was an association between the COVID-19 pandemic and decreased sexual activity, especially in terms of frequency of sexual intercourse in both women and (to a less extent) men. Based on the findings of a Spanish study, during the COVID-19 confinement, 71.3% of the population (*N* = 382) reported to be engaged in sexual activity at least once per week on average and were thus classified as sexually active. It was also concluded that confinement may not have strongly influenced the sexual activity [93]. However, the findings of a study on the British people’s sexual activity during COVID-19 showed that 60.1% of the study sample reported not being sexually active during self-isolation/social distancing [37]. This discrepancy in the findings of studies on sexual activity during the COVID-19 pandemic may be due to cultural differences in sexual activity patterns among different communities as well as the methods of studies. In addition, the physical contact of partners during sexual intercourse increases the possibility of transmission of infection; therefore, this could be one of the reasons for the decrease in sexual activity during the COVID-19 pandemic.

The still ongoing COVID-19 outbreak is currently the biggest challenge for the health systems worldwide, imposing a significant mortality and morbidity rate. As sexual activity plays a key role in physical and mental health and, especially, in improving the immune system, the present study identifies and recommends that the factors that reduce sexual functioning and activity should be taken into account by health policy- and decision-makers in order to promote sexual and physical health that would ultimately help prevent and control the individual and societal consequences of the COVID-19 pandemic.

Since it can be anticipated that in the future further outbreaks and pandemics are highly likely to occur again, public health workers and decision- and policymakers should be aware that COVID-19 related restrictions can significantly impair sexual functioning and activity.

## Limitations

This systematic> review study has some limitations that should be noted. Only few studies utilized standardized tools and questionnaires like the FSFI or IIEF-15, which influenced the number of studies that could be included and synthesized in the present meta-analysis. Moreover, the current meta-analysis only assessed heterosexual people, so the results may only be generalized to the heterosexual population and not to other individuals/populations with different sexual orientation and gender identity. A further shortcoming is represented by the high amount of heterogeneity among studies: the cultural, societal, and ethnic differences among the various participants and sample sizes could, at least, partially, explain this. The COVID-19 pandemic can affect the sexual lives in different countries in different ways. Sexual satisfaction is generally higher in men than women. Moreover, we limited our eligible studies to those written in English.

## Conclusion

The results of the present study showed that COVID-19 related restrictions were correlated with higher rates of sexual dysfunction and reduced sexual activity. This outbreak has been able to affect the quality of both women’ and men’s sexual life. Sex is one of the dimensions of every person’s life. Researchers should identify the factors that lead to sexual dysfunction due to COVID-19 in their community, and sex researchers should design and implement effective programs and interventions to reduce the burden of sexual dysfunction, given the psychological strain that the COVID-19 pandemic puts on individuals.

## Supplementary Information

**Additional file 1.**

## Abbreviations

WOS: Web of Science
SMD: Standardized Mean Difference
FSFI: Female Sexual Function Index
IEEF-5: International Index of Erectile Function-5 items
PEDT: Premature Ejaculation Diagnostic Tool
PRISMA: Preferred Reporting Items for Systematic Reviews and Meta-Analyses
CI: Confidence interval
